# [^18^F]FDG PET/CT Signal Correlates with Neoangiogenesis Markers in Patients with Fibrotic Interstitial Lung Disease Who Underwent Lung Biopsy: Implication for the Use of PET/CT in Diffuse Lung Diseases

**DOI:** 10.2967/jnumed.123.266445

**Published:** 2024-04

**Authors:** Joanna C. Porter, Balaji Ganeshan, Thida Win, Francesco Fraioli, Saif Khan, Manuel Rodriguez-Justo, Raymond Endozo, Robert I. Shortman, Luke R. Hoy, Toby M. Maher, Ashley M. Groves

**Affiliations:** 1 CITR, UCL Respiratory, University College London, London, United Kingdom;; 2 Interstitial Lung Disease Centre, University College London Hospital, London, United Kingdom;; 3 Institute of Nuclear Medicine, University College London and University College London Hospital, London, United Kingdom;; 4 Lister Hospital, North East Herts Trust, Stevenage, United Kingdom;; 5 Research Department of Pathology, University College London, and Department of Histopathology, University College London Hospital, London, United Kingdom; and; 6 Keck School of Medicine, University of Southern California, Los Angeles, California

**Keywords:** [^18^F]FDG PET/CT, ILD, fibrosis, angiogenesis, microvessel

## Abstract

The use of [^18^F]FDG PET/CT as a biomarker in diffuse lung diseases is increasingly recognized. We investigated the correlation between [^18^F]FDG uptake with histologic markers on lung biopsy of patients with fibrotic interstitial lung disease (fILD). **Methods:** We recruited 18 patients with fILD awaiting lung biopsy for [^18^F]FDG PET/CT. We derived a target-to-background ratio (TBR) of maximum pulmonary uptake of [^18^F]FDG (SUV_max_) divided by the lung background (SUV_min_). Consecutive paraffin-embedded lung biopsy sections were immunostained for alveolar and interstitial macrophages (CD68), microvessel density (MVD) (CD31 and CD105/endoglin), and glucose transporter 1. MVD was expressed as vessel area percentage per high-power field (Va%/hpf). Differences in imaging and angiogenesis markers between histologic usual interstitial pneumonia (UIP) and non-UIP were assessed using a nonparametric Mann–Whitney test. Correlation of imaging with angiogenesis markers was assessed using the nonparametric Spearman rank correlation. Univariate Kaplan–Meier survival analysis assessed the difference in the survival curves for each of the angiogenesis markers (separated by their respective optimal cutoff) using the log-rank test. Statistical analysis was performed using SPSS. **Results:** In total, 18 patients were followed for an average of 41.36 mo (range, 5.69–132.46 mo; median, 30.07 mo). Only CD105 MVD showed a significantly positive correlation with [^18^F]FDG TBR (Spearman rank correlation, 0.556; *P* < 0.05, *n* = 13). There was no correlation between [^18^F]FDG uptake and macrophage expression of glucose transporter 1. CD105 and CD31 were higher for UIP than for non-UIP, with CD105 reaching statistical significance (*P* = 0.011). In all patients, MVD assessed with either CD105 or CD31 quantification on biopsy predicted overall survival. Patients with CD105 MVD of less than 12 Va%/hpf or CD31 MVD of less than 35 Va%/hpf had a significantly better prognosis (no deaths during follow-up in the case of CD105) than did patients with higher scores of CD105 MVD (median survival, 35 mo; *P* = 0.041, *n* = 13) or CD31 MVD (median survival, 28 mo; *P* = 0.014, *n* = 13). **Conclusion:** Previous work has used [^18^F]FDG uptake in PET/CT as a biomarker in fILD. Here, we highlight a correlation between angiogenesis and [^18^F]FDG TBR. We show that MVD is higher for UIP than for non-UIP and is associated with mortality in patients with fILD. These data set the scene to investigate the potential role of vasculature and angiogenesis in fibrosis.

## Visual Abstract

**Figure:**
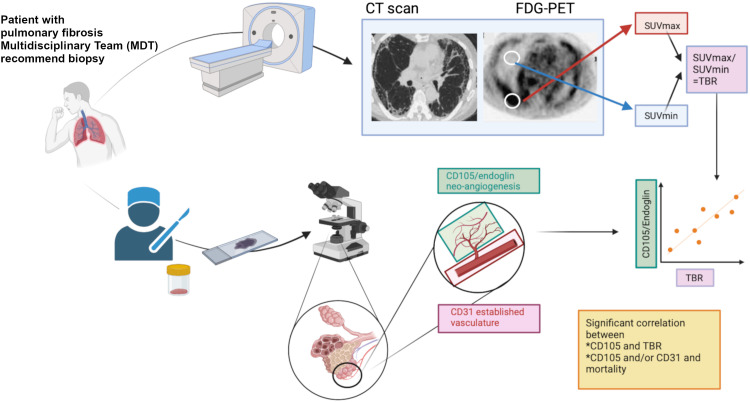

There is an urgent clinical need for better biomarkers in interstitial lung disease (ILD) for both patient management and as surrogate endpoints for clinical trials (1). The potential role of PET/CT in guiding the management of patients with ILD has been recognized for some time (2). We have previously shown that [^18^F]FDG uptake on PET/CT is a prognostic biomarker in idiopathic pulmonary fibrosis (IPF) that can improve the ability of the clinical sex, age, and physiology score to predict outcomes (3). Determining the basis of this signal may improve the clinical utility of [^18^F]FDG PET/CT through patient stratification, the assessment of treatment response, and potentially valuable mechanistic and therapeutic insights into fibrotic ILD (fILD).

Vessel volume and vessel density, as determined by tissue-density measurements on CT imaging, correlate positively with both physiologic measures (4) and outcomes in IPF (5). Unfortunately, the prognostic value of lung vascularity, reflected by either microvessel density (MVD) or its correlation with [^18^F]FDG uptake, has not been explored, and there remains contention over whether there is a paucity or an excess of blood vessels in the fibrotic lung (6).

In this study, we investigated the hypothesis that MVD and macrophage phenotype/glucose transporter 1 (GLUT-1) expression on immunohistochemistry were potential predictors of mortality in fILD and examined the correlation between [^18^F]FDG uptake on PET imaging and angiogenesis and macrophage subsets for different histologic presentations of disease (usual interstitial pneumonia [UIP] vs. non-UIP).

## MATERIALS AND METHODS

### Patients

This prospective, single-center study was approved by the London-Harrow Research Ethics Committee (reference 06/Q0505/22), and all participants signed an informed consent form. In total, 18 patients were included in the study on the basis of having a radiologic diagnosis of fILD and either having had or being scheduled to have a clinically required biopsy of an area of lung determined radiographically. Patients were excluded if they had ongoing inflammatory or malignant disease or had been on treatment (immunosuppression or antifibrotics) for ILD in the previous 3 mo. Each participant had an [^18^F]FDG PET/CT scan that was performed an average of 184 ± 692 d after the biopsy. Pulmonary function tests were undertaken in all patients where possible and quantified as forced vital capacity and transfer factor of the lung for carbon monoxide.

### PET/CT Image Acquisition

The methodology we used is described in detail elsewhere (7–10). All images were acquired on the same PET/CT scanner (VCT PET/64-detector CT instrument; GE Healthcare). Participants were each injected with 200 MBq of [^18^F]FDG and allowed to rest peacefully in a private cubicle during the 1-h uptake time. The participants were positioned supine on the CT table with their arms held above their head and were instructed to keep still throughout the procedure, which took approximately 20 min. The first scan was a CT attenuation-correction scan of the thorax, immediately followed by a PET emission scan (8 min per bed position) with identical anatomic coverage. The final scan was a high-resolution CT scan of the lungs that was performed on a deep inspiratory breath-hold, using the following CT parameters: 64 × 1.25 mm detectors, a pitch of 0.53, and a 1.25-mm collimation (120 kVp and 100 mAs).

### PET/CT Image Analysis

PET images were analyzed by a dual-trained nuclear medicine physician and a nuclear medicine technologist with more than 5 y of experience in quantifying pulmonary uptake in [^18^F]FDG PET/CT imaging of ILD. CT images were reviewed independently of the PET/CT analysis by a dedicated thoracic radiologist.

All images were loaded onto an ADW workstation (GE Healthcare). All datasets underwent image processing that has been previously described in detail and has been shown to have high inter- and intraobserver reproducibility (2). The area of most intense pulmonary [^18^F]FDG uptake was identified visually, and then a 2-dimensional region of interest was drawn over the area and the highest pixel value (SUV_max_) measured.

The region of pulmonary parenchyma with the lowest uptake (SUV_min_) was similarly identified and confirmed by the dedicated thoracic radiologist to be morphologically normal lung parenchyma on coregistered CT. The SUV_min_ was considered a measure of the background lung uptake and was used to calculate the lung target-to-background ratio (TBR = SUV_max_/SUV_min_) (2).

### Histologic Image Analysis

**FIGURE 1.**
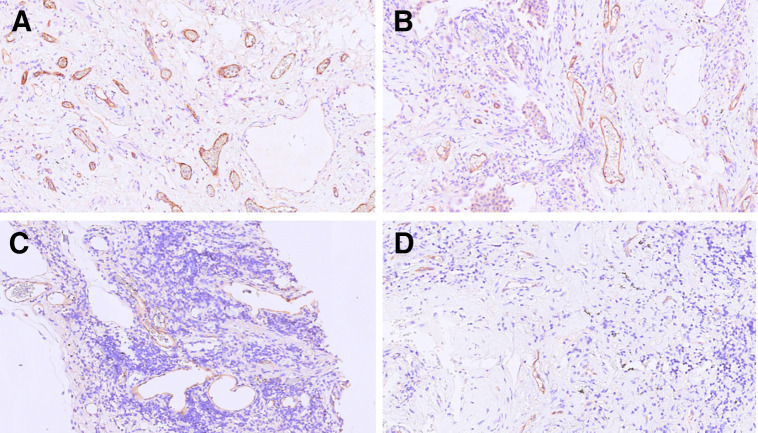
Immunohistochemical images of CD105 staining of microvessels in UIP (A and B) and non-UIP (C and D) at ×200 magnification (digital-based platform).

Biopsy samples were taken from affected lung at the discretion of the thoracic surgeon or bronchoscopist, but biopsies were not specifically targeted to PET-avid areas when that information was available. Immunostaining was performed for CD31, a panendothelial cell marker expressed on mature and immature vessels; CD105/endoglin, a proliferation-related endothelial cell marker that is more specific for new immature vessels (11); CD68/CD80 for M1-like macrophages; and CD68/CD163 for M2-like macrophages and GLUT-1 expression. Consecutive paraffin-embedded sections obtained from biopsy were immunostained for CD68 (514H12; Leica Biosystems) (alveolar and interstitial macrophages), CD31 (1A10; Novocastra) (panendothelial marker), CD105 (4G11; Novocastra) (endoglin, a protein expressed in angiogenic endothelial cells), and GLUT-1 (polyclonal; Millipore) (the receptor for glucose uptake). Quantification of CD31, CD105, and GLUT-1/CD68 was performed by a single observer (with >20 y of experience) masked to the [^18^F]FDG PET/CT imaging findings through a semiquantitative analysis of immunoreactivity of the markers. For CD31 and CD105, four 1.060 mm^2^ fibrous areas of highest vascularization (hot spots) were counted at a ×20 magnification on an Olympus BX51 microscope (12). Figure 1 shows immunohistochemical images of CD105 staining of microvessels in UIP (Figs. 1A and 1B) and non-UIP (Figs. 1C and 1D) at a ×200 magnification (digital-based platform).

**FIGURE 2.**
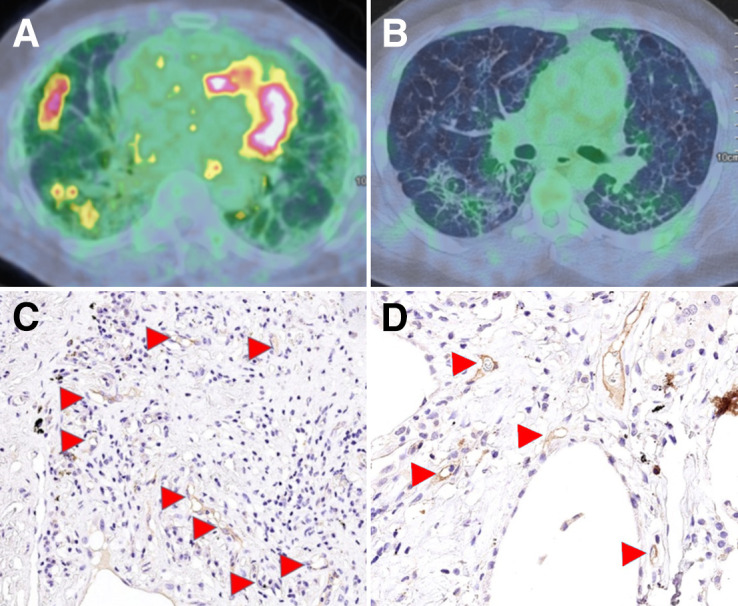
Representative participant [^18^F]FDG PET/CT scans showing high (A) and low (B) TBR of [^18^F]FDG uptake. Immunohistochemistry images of stained patient biopsies show high (C) and low (D) expression of CD105/endoglin (arrowheads).

CD31 and CD105 are expressed as vessel area percentage per high-power field (Va%/hpf); GLUT-1 staining of CD68 macrophages was scored as absent (0), intermediate (1), or high (2) (8). CD68 macrophages were also costained for CD80 as a marker of M1-like macrophages and CD163 to distinguish the M2-like subtypes. Figure 2 shows typical examples of histologic staining and [^18^F]FDG PET/CT scans in different participants.

### Follow-up

The follow-up period was defined as the period between the date of the participant’s [^18^F]FDG PET/CT scan and the date of the patient’s death or of the patient’s last living contact with medical services, calculated in months.

### Statistical Analysis

Statistical analysis was performed using SPSS (version 27.0; IBM Corp.), with a *P* value of less than 0.05 considered to be significant. Differences in imaging and angiogenesis markers between the histology were assessed using a nonparametric Mann–Whitney test. Univariate Kaplan–Meier survival analysis assessed the difference in the survival curves for each of the angiogenesis markers (separated by their respective optimal cutoff) using the log-rank test. The statistical methodology for determining the optimal cutoff (maximum log-rank or minimum *P* value) in univariate survival analysis was performed as previously reported (13). This methodology has previously been used in prognostication of IPF patients (3). Correlation between the imaging and angiogenesis markers was assessed using nonparametric Spearman rank correlation.

## RESULTS

In total, 18 patients with fILD were followed for an average of 41.36 mo (range, 5.69–132.46 mo; median, 30.07 mo). Histologic information was available for all 18 participants, of whom 72% (13/18) had histologic UIP and met the clinical criteria for IPF (14) and 28% (5/18) had non-UIP findings, of which 4 were considered fibrotic nonspecific interstitial pneumonia and 1 was thought to be fibrotic organizing pneumonia. Technical factors, mainly due to insufficient tissue volume, prevented CD105 staining in 5 of 18 cases. Table 1 illustrates the pulmonary function test results, histology, treatment, neoangiogenesis, vasculature, macrophages, and PET uptake markers for each patient in the study population.

**TABLE 1.** Participant Characteristics, Immunohistochemistry Results, and [^18^F]FDG PET/CT Quantification*

Patient	Forced vital capacity prediction (%)	Transfer factor of lung for carbon monoxide prediction (%)	Histology	Treatment for ILD	CD105 (Va%/hpf)	CD31 (Va%/hpf)	GLUT-1 macrophage score	TBR	CD80 average of hpf counts	CD163 average of hpf counts	CD163:CD80 ratio
1	65	57	UIP	No	22	26	0	5.29	42.5	57.5	1.35
2	54	27	NSIP	No	19	23	1	7.71	NA	NA	NA
3	47	33	OP	No	10	15	1	3	NA	NA	NA
4	57	29	NSIP	No	10	34	0	4.6	16.5	75	4.55
5	99	79	UIP	No	23	30	0	4.29	9.5	34	3.58
6	73	41	UIP	No	44	NA	0	4.67	1.5	31.5	21
7	NA	45	UIP	No	NA	NA	2	6	2.5	58.5	23.4
8	95	64	UIP	No	18	38	0	3.86	32.5	52.5	1.62
9	91	66	NSIP	No	NA	29	1	5.29	33	36	1.09
10	77	47	NSIP	No	14	NA	0	3.33	20.5	51	2.49
11	104	70	UIP	No	25	NA	1	4.6	4.5	85	18.89
12	99	63	UIP	No	34	70	0	5.86	18.5	65	3.51
13	75	54	UIP	No	17	21	0	3.75	10.5	56	5.33
14	99	NA	UIP	No	NA	55	2	6.33	7.5	31.5	4.2
15	43	43	UIP	No	NA	45	0	5.17	3	51.5	17.17
16	63	63	UIP	No	24	35	0	10.29	1	42.5	42.5
17	NA	NA	UIP	No	NA	NA	2	6.17	NA	NA	NA
18	58	40	UIP	No	19	45	1	12	11.5	38	3.3

* Lung function results were within 10 d of [^18^F]FDG PET/CT scan.

NSIP = nonspecific interstitial pneumonia; NA = not available; OP = organizing pneumonia.

**FIGURE 3.**
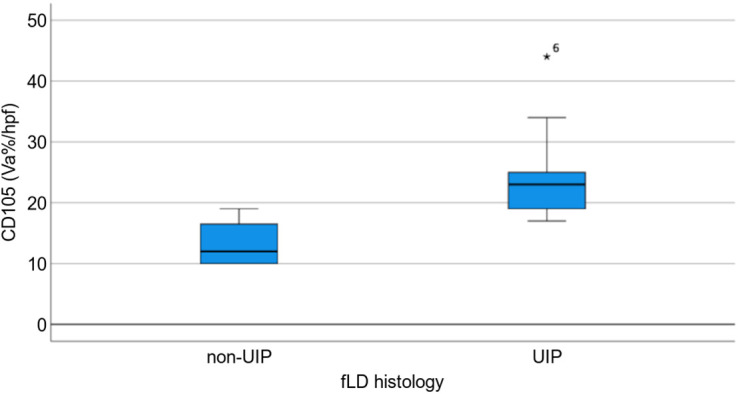
Box plot highlighting increased distribution of CD105 in UIP vs. non-UIP histology (*P* = 0.011). *Patient 6 outlier (CD105 = 44). fLD = fibrotic lung disease.

On biopsy, only CD105 was significantly higher (*P* = 0.011; Fig. 3) for UIP (*n* = 9) than for non-UIP (*n* = 4). CD31 was higher for UIP histology but did not reach statistical significance (*P* = 0.08, *n* = 13).

**FIGURE 4.**
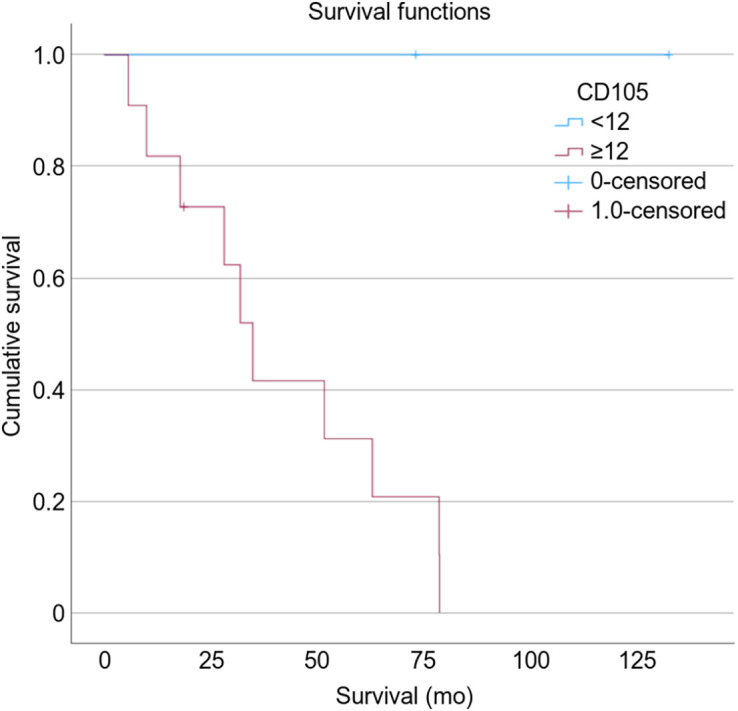
Kaplan–Meier survival curve showing how neoangiogenic marker CD105 predicts overall survival (*P* = 0.041).

**FIGURE 5.**
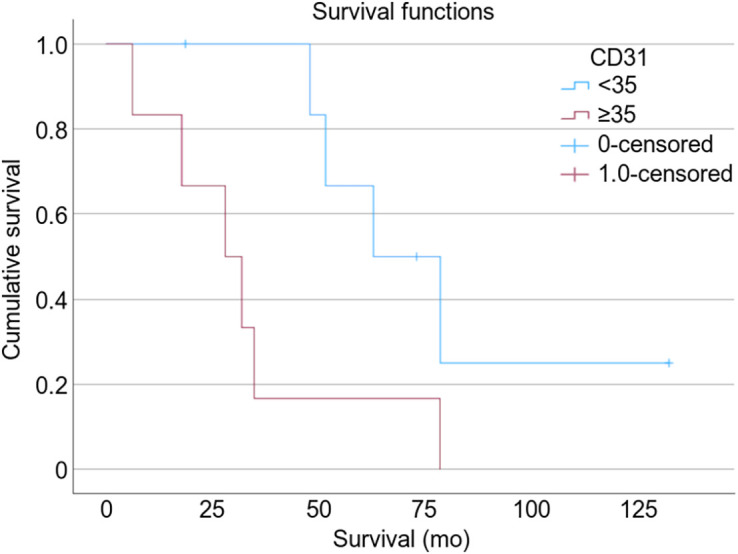
Kaplan–Meier survival curve showing how vasculature marker CD31 predicts overall survival (*P* = 0.014).

In all participants, both CD105 MVD and CD31 MVD staining on biopsy predicted overall survival in fILD patients. Patients with a CD105 MVD of less than 12 Va%/hpf had no deaths during their follow-up, and patients with a CD31 MVD of less than 35 Va%/hpf had improved survival than that seen with a higher CD31 MVD. The median survival of patients with a CD105 MVD of at least 12 Va%/hpf and a CD31 MVD of at least 35 Va%/hpf was 35 mo (*P* = 0.041, *n* = 13; Fig. 4) and 28 mo (*P* = 0.014, *n* = 13; Fig. 5), respectively. There was no correlation between macrophage numbers, M1 or M2 subtypes, or M1:M2 ratios with survival.

**FIGURE 6.**
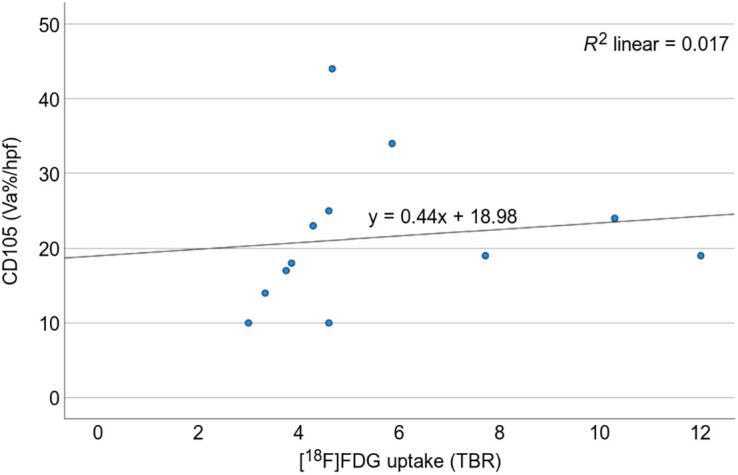
Scatterplot demonstrating positive correlation (Spearman rank correlation, 0.556; *P* < 0.05) between CD105 and [^18^F]FDG uptake (TBR).

The [^18^F]FDG PET/CT signal in the 13 participants with UIP histology and IPF was found to be similar to that of the 5 participants with non-UIP histology, with no significant difference in TBR (*P* = 0.29). However, the TBR did have a significant positive correlation with the angiogenesis marker CD105 MVD (Spearman rank correlation, 0.556; *P* < 0.05, *n* = 13; Fig. 6). The CD31 MVD was not significantly correlated with TBR (Spearman rank correlation, 0.479; *P* = 0.097, *n* = 13), and there was no correlation between [^18^F]FDG uptake and either macrophage expression of GLUT-1 or macrophage subsets.

## DISCUSSION

In this study, we found CD105 MVD as a marker of neoangiogenesis that was significantly correlated positively with mortality, UIP histology, and [^18^F]FDG TBR in patients with fILD. CD31, a marker of established vasculature, significantly correlated positively with mortality but not with PET signal or histology. We also found no correlation between GLUT-1 expression on macrophages and [^18^F]FDG PET/CT or with macrophage subsets, as determined by CD68 (M1) and CD103 (M2).

We have previously shown using cross-validation in a cohort of 113 patients that TBR derived from [^18^F]FDG PET/CT is predictive of the outcome in patients with IPF (3) and that a high percentage of vessels on quantitative CT, measured as pulmonary vessel volume (4) or as vessel percentage (5), in relatively unaffected parts of the lung enables identification of IPF patients who have worse health outcomes. Although the biologic basis for this is unclear, Jacob et al. (4) offered 3 plausible explanations: blood-flow diversion from advanced fibrotic areas to relatively spared lung regions; a dilatation effect on blood vessels due to increased negative inspiratory pressure, secondary to increased lung stiffness; and the effect of pleuroparenchymal and bronchopulmonary arterial anastomosis. To reconcile these findings, we suggest that, despite active neoangiogenesis in fibrotic areas, these vessels may not significantly contribute to blood flow.

Angiogenesis may be driven by hypoxia and hypoxia-inducible factor transcriptional regulators, but our previous work shows very little hypoxia-inducible factor activation in the IPF lung (9). Transforming growth factor-β is a key cytokine in fibrosis and angiogenesis, and endoglin/CD105 acts as a transforming growth factor-β coreceptor to bind leucine-rich glycoprotein 1 and drive aberrant vessel formation during neoangiogenesis (15).

GLUT-1, the main transporter for [^18^F]FDG, is found in erythrocytes and alveolar macrophages in the fibrotic lung, which has led to the suggestion that the [^18^F]FDG PET/CT signal in ILD may reflect inflammation or neoangiogenesis (16). Macrophages play a role in ILD, with monocyte-derived macrophages contributing to fibrotic potential, and can be broadly considered to exist on a spectrum of inflammatory (M1-like) or antiinflammatory and fibrotic (M2-like) behavior, dependent on their cell surface molecule expression and cytokine production profiles (17). However, there are inconsistencies with this view, and a recent attempt has been made to provide a consensus classification (18). In general, M1-like or classically activated macrophages show enhanced glycolysis and GLUT-1 expression (19) and are thought to be elevated during inflammation, with M2-like or alternatively activated macrophages increased in the fibrotic stage of disease. This view has given rise to the targeting of M2-like macrophages as a possible treatment strategy in IPF (20). Distinguishing these populations on immunohistochemistry is difficult (21), and with these limitations in mind, we used CD80 and CD163 surface staining to distinguish the M1-like (CD68-positive/CD80high/CD163low) and M2-like (CD68-positive/CD80low/CD163high) phenotypes, respectively. Although we found no differences in [^18^F]FDG PET uptake parameters with differing macrophage populations on immunohistochemistry, further study may be warranted using more specific markers of transcription and other factors (21).

Although our study population was limited in size, only a small minority of ILD patients have lung biopsies, and therefore, our cohort of 18 fILD patients with matched [^18^F]FDG PET/CT images and immunohistochemistry is the largest reported to the best of our knowledge. Unfortunately, it was difficult to control the time between the biopsy and the [^18^F]FDG PET/CT scan, causing a large variation in the biopsy–scan interval, which is a limitation of this study. In addition, the association between MVD and survival in such a small group is open to valid criticism. Although the determination of optimal cutoff points in our univariate survival analysis followed a statistically recognized methodology previously reported (13), it is important to note that this is a pilot study and future studies should include robust validation procedures to confirm and generalize the findings. Although perfect registration between the biopsy location and PET measurements was not possible, it is less problematic in diffuse lung disease than with focal lesions such as lung cancer. Using a volume of interest near the biopsy location rather than a 2-dimensional region of interest for PET uptake measurements may be a better reflection of the underlying lung biology and could be the subject of a future study, but it is the inherent heterogeneity of both immunochemistry and radiologic appearance that makes quantification of ILD difficult. This challenge is being increasingly met through computer-aided quantitative analysis, and CT texture has already been shown to be a significant predicator of IPF mortality that is independent of lung parenchymal patterns (22). It is plausible that CT texture analysis could provide useful information about pulmonary vasculature and PET uptake in the future.

## CONCLUSION

Previous work has used [^18^F]FDG PET/CT as a biomarker for guiding personalized treatment and prognostication in IPF, and its utility is increasingly recognized in other conditions such as in post–coronavirus disease 2019 lung disease (10). Understanding the basis of the [^18^F]FDG PET/CT signal may offer mechanistic insights into ILD and identify novel pharmacologic targets around fibrosis. In this study, we highlight a marker of neoangiogenesis and one of established vasculature as a predictor of mortality and possible associations between neoangiogenesis and [^18^F]FDG uptake (TBR) and histology in patients with fILD. Whether such positive correlations reflect a direct causal link is unknown, but they do set the scene to investigate the potential role of vasculature and angiogenesis in fibrosis.

## DISCLOSURE

Joanna Porter was funded by a Medical Research Council new investigator award and by Breathing Matters; this work was undertaken at UCLH/UCL, which received a proportion of funding from the U.K.’s Department of Health’s National Institute of Health Research Biomedical Research Centres’ funding scheme. No other potential conflict of interest relevant to this article was reported.

## Notes

KEY POINTS **QUESTION:** Does the lung uptake of [^18^F]FDG on PET/CT correlate with histologic markers in patients with fILD? **PERTINENT FINDINGS:** This study revealed a positive correlation between lung uptake of [^18^F]FDG and MVD (CD105 MVD) in fILD patients. Higher CD105 MVD in UIP cases than in non-UIP cases was associated with increased mortality. **IMPLICATIONS FOR PATIENT CARE:** The findings emphasize the potential significance of angiogenesis, particularly CD105 MVD, in fILD, suggesting avenues for further research and therapeutic exploration.
